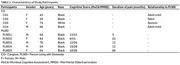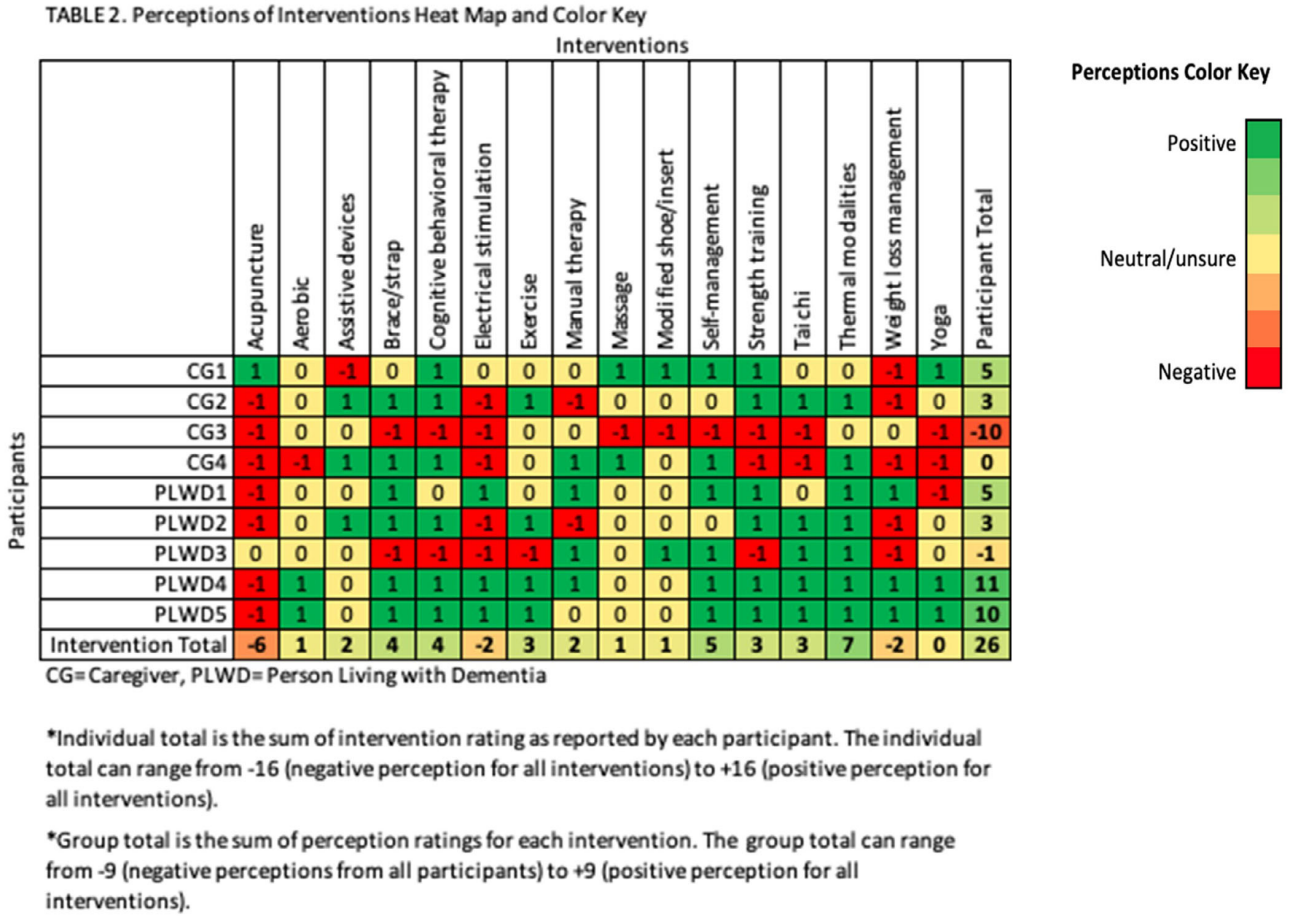# Navigating Pain Management: Perspectives of Community‐Dwelling People with Dementia and their Caregivers on Nonpharmacological Interventions

**DOI:** 10.1002/alz70858_107386

**Published:** 2025-12-26

**Authors:** Amy Kwok, Ben Senderling, Justine S. Sefcik, Annalisa Na

**Affiliations:** ^1^ Drexel University, Philadelphia, PA, USA

## Abstract

**Background:**

For pain management there are 16 non‐pharmacological interventions, ranging from acupuncture to yoga, reviewed in clinical practice guidelines (CPGs) endorsed by the American Academy of Orthopedic Surgeons (AAOS), American Geriatrics Society (AGS), and American College of Rheumatology (ACR). Since dementia does not impact underlying pain mechanisms, clinicians can recommend these interventions to community‐dwelling persons living with dementia (PLWD) with chronic pain and their caregivers. However, it is unknown how PLWD and their caregivers perceive these interventions. Learning about these perceptions can provide insight into factors influencing engagement and outcomes in pain management. Therefore, this study explored the perspectives of community‐dwelling PLWD and their caregivers on nonpharmacological pain management interventions.

**Method:**

We took a qualitative descriptive approach involving semi‐structured interviews. Eligible PLWD were: ≥ 60 years old, cognitively impaired indicated by Montreal Cognitive Assessment ≤ 16, pain duration ≥ 3 months with intensity of ≥ 3/10. Eligible caregivers provided regular care to PLWD ≥ 1 year. Interviews followed a guide outlining the 16 interventions from AAOS, AGS, and ACR CPGs. An initial content analysis led us to code PLWD and caregiver perceptions of interventions as: positive (+1) for perceived benefits, neutral/unsure (0) for uncertainty or lack of familiarity, and negative (‐1) for concerns or reluctance to use the intervention. These perceptions were visualized in a heatmap to display high and low thematic density.

**Result:**

Nine participants were interviewed: five PLWD and four caregivers (Table 1). Heatmap visualization (Table 2) illustrated intensity scores of thermal modalities (+7) and self‐management (+5) as most positively perceived for pain management. While acupuncture (‐6), electrical stimulation (‐2) and weight loss management (‐2) were most negatively viewed. Interventions that the majority were unsure of included aerobic exercise (*n* = 6), massage (*n* = 6), modified shoes/inserts (*n* = 6) and assistive devices (*n* = 5).

**Conclusion:**

Perceptions of nonpharmacological pain management varied among PLWD and caregivers. Thermal modalities and self‐management were most favorable, while several other interventions were not. Uncertainty about some interventions for chronic pain suggests a need for patient education. In clinical practice, interventions should be tailored and aligned with preferences while addressing misconceptions of the PLWD and caregivers.